# Stigma gets in my way: Factors affecting client-provider communication regarding childbearing among people living with HIV in Uganda

**DOI:** 10.1371/journal.pone.0192902

**Published:** 2018-02-20

**Authors:** Jolly Beyeza-Kashesya, Rhoda K. Wanyenze, Kathy Goggin, Sarah Finocchario-Kessler, Mahlet Atakilt Woldetsadik, Deborah Mindry, Josephine Birungi, Glenn J. Wagner

**Affiliations:** 1 Mulago Hospital Department of Obstetrics and Gynaecology, Makerere University College of Health Sciences, Kampala, Uganda; 2 Department of Disease Control and Environmental Health, Makerere University School of Public Health, College of Health Sciences, Kampala, Uganda; 3 Health Services and Outcomes Research, Children's Mercy Hospitals and Clinics, Schools of Medicine and Pharmacy, University of Missouri–Kansas City, Missouri, United States of America; 4 University of Kansas Medical Center, Department of Family Medicine, Kansas City, Missouri, United States of America; 5 RAND Corporation, Santa Monica, California, United States of America; 6 University of California, Los Angeles Center for Culture and Health, Los Angeles, California, United States of America; 7 The AIDS Support Organization, Kampala, Uganda; British Columbia Centre for Excellence in HIV/AIDS, CANADA

## Abstract

**Introduction:**

Many HIV-affected couples living in sub-Saharan Africa desire to have children, but few quantitative studies have examined support for their childbearing needs. Our study explored client-provider communication about childbearing and safer conception among HIV clients in Uganda.

**Methods:**

400 Ugandan HIV clients in committed relationships and with intentions to conceive were surveyed. Knowledge, attitudes and practices related to childbearing, and use of safer conception methods were assessed, including communication with providers about childbearing needs, the correlates of which were examined with bivariate statistics and logistic multivariate analysis.

**Results:**

75% of the sample was female; 61% were on antiretroviral therapy; and 61% had HIV-negative or unknown status partners. Nearly all (98%) reported the desire to discuss childbearing intentions with their HIV provider; however, only 44% reported such discussions, the minority (28%) of which was initiated by the provider. Issues discussed with HIV providers included: HIV transmission risk to partner (30%), HIV transmission risk to child (30%), and how to prevent transmission to the child (27%); only 8% discussed safer conception methods. Regression analysis showed that those who had communicated with providers about childbearing were more likely to have been diagnosed with HIV for a longer period [OR (95% CI) = 1.09 (1.03, 1.15)], while greater internalized childbearing stigma was associated with lower odds of this communication [OR (95% CI) = 0.70 (0.49, 0.99)], after controlling for all bivariate correlates and basic demographics.

**Conclusions:**

Communication between HIV clients and providers about childbearing needs is poor and associated with stigma. Innovations to mitigate stigma among clients as well as training to improve health worker communication and skills related to safer conception counseling is needed.

## Introduction

Major strides have been achieved in HIV prevention among HIV-affected couples through advocacy for consistent condom use [1] and use of antiretroviral therapy [2] However, in Uganda up to 60% of people living with HIV (PLHIV) desire to have a child [3–6] and one third of discordant couples have gone ahead to produce children [7]; albeit without the assistance of safer conception practices [8, 9].

In resource-limited settings like Uganda, the dilemma between wanting to have children and limiting risks of transmitting HIV (if partner is also HIV-positive) is common among people living with HIV (PLHIV) [3, 7]. In some couples, the desire to have children sometimes overrides fears of transmission, and they practice unprotected sex in an attempt to conceive [7]. In addition, several studies have reported that many couples are not mutually aware of their HIV status [10, 11]. This limits the use of available opportunities of safer conception methods to prevent HIV transmission.

Globally, there has been an increase in awareness of the reproductive rights of PLHIV and the need to promote use of safer conception methods [12–14]; however, due to lack of guidelines and provider training, provision of safer conception counseling is not yet a standard component of health services in Sub Saharan Africa [15]. The use of antiretroviral therapy is known to markedly reduce HIV transmission if suppression is in undetectable levels [16–19] however uptake of and adherence to ART is often suboptimal [20]. In addition, male circumcision [21] has been rolled out in most health care facilities and could act as an added protective method, although its coverage remains very low in most high prevalence countries in sub-Saharan Africa and thus the for need additional methods to reduce transmission risk. Other safer conception methods that are specific to the context of conception and inexpensive (feasible in low resource settings) include Pre-exposure prophylaxis (PrEP), sexually transmitted disease treatment, manual self-insemination, and timed unprotected intercourse during ovulation [13, 22], which reduce the frequency of HIV exposure by limiting unprotected coital acts. However, despite the widespread demand for safer conception services as a global strategy to reduce HIV incidence, the majority of PLHIV need assistance from health workers to understand and effectively use these safer conception methods, including identifying the timing of ovulation [20]. Some studies have reported low self-efficacy to providing SCC among HIV-providers [8, 23, 24], and therefore, HIV clients and their providers often do not discuss the client’s childbearing desires or plans prior to pregnancy [25].

Research from sub-Saharan Africa, including Uganda, shows that only 20–40% of clients with fertility intentions discuss these intentions with their HIV providers [25–29]. Up-to-date, childbearing stigma among both the providers and clients muffles the content and depth of discussions about safer conception practices [27, 30–32]. Historically, HIV infection was viewed as a terminal disease; therefore providers openly discouraged clients from having children for fear of high mortality risks but also to prevent HIV transmission [24, 27, 30–34]. Most HIV programs largely focused on preventing new infections as a core component of HIV care; therefore emphasizing adherence to condom use. After 2001, the US Centers for Disease Control and Prevention (CDC) encouraged providers to offer information and support to HIV-affected couples to explore their reproductive options [35]. Nonetheless, provider judgmental attitudes that increase stigmatization of childbearing continued [27, 30–32]; manifesting in different forms such as failure to provide reproductive health services or coercing PLHIV to accept sterilization [36]. While infringement on the reproductive health rights of PLHIV through coerced sterilization has attracted lawsuits against such health providers [37], sterilization of HIV-positive women at caesarian section delivery continues to be reported [38]. Such practices could create mistrust among PLHIV and deter them from initiating childbearing discussions with providers. Therefore, on the one hand, clients avoid talking with providers due to perceived provider stigma and their own internalized stigma [34, 39–41], on the other hand, low self-efficacy to provide SCC among HIV-providers (often interpreted by patients as denial of service) contributes to the perceived stigma by clients [23, 24, 42].

In Uganda, there is a challenge because reproductive health counseling for PLHIV mostly focuses on contraception and preventing pregnancy [26]. Limited quantitative information exists on whether and how HIV care providers discuss and offer support for the sexual and reproductive health needs of clients who seek childbearing, including the use of safer conception methods. This paper explored the process of initiation of the discussion and correlates of client-provider communication about childbearing and safer conception among HIV clients who desire to have children in Uganda.

## Materials and methods

### Study setting

The study was conducted at The AIDS Support Organization (TASO) HIV care and treatment sites in Kampala and Jinja, Uganda. TASO is a non-governmental organization founded in 1987 to provide care and support for Ugandans who are either living with or affected by HIV/AIDS. The Kampala site is located next to the Mulago National Referral Hospital and has over 6700 active clients. The Jinja site is located within the Jinja Regional Referral Hospital campus and provides HIV care to over 8000 clients as at June 2016. In addition to ART and counseling services, TASO has well-established family planning and contraception services at its clinics, but has not integrated the routine delivery of safer conception services.

### Participants

Clients at the two study clinics were eligible for the study if they were (1) 18 years or older, (2) married or in a committed heterosexual relationship, and (3) reported an intention to conceive a child with their partner within the next 24 months. Only one member of a couple was allowed to participate to ensure the participants were independent of each other. Partner HIV status was not a part of the eligibility criteria since safer conception methods are also relevant to HIV sero-concordant couples for the purpose of limiting risks of super-infection and transmission of resistant virus. The cohort was recruited between May and October of 2013. Recruitment took place primarily during the triage phase when clients registered their attendance at clinic visits. A brief screening was conducted with adult clients by the triage personnel. Those who were likely eligible and expressed interest were referred to the research coordinator for a more thorough screening and consent procedures.

After providing written informed consent, participants were administered the baseline survey questionnaire at the clinic premises after the provider consultation and care. Follow-up surveys were scheduled at 6-month intervals for 24 months, or until the participant (or their partner) became pregnant in which case their participation ended after a post-delivery survey was completed. We present analysis from the baseline data. Participants received 15,000 Ush ($6 USD) for completing each survey. The study protocol was approved by the Institutional Review Boards at Makerere University School of Biomedical Sciences Research and Ethics Committee, RAND, TASO, and the Uganda National Council of Science and Technology.

### Measures

All measures were translated (using standard forward and back translation methods) into and administered in Luganda, the most common native language in the study setting. Trained and experienced interviewers used computer-assisted personal interview software to administer the survey.

#### Demographics

These included: age, sex, education level (whether or not any secondary education had been completed), and monthly income.

#### Reproductive health history and current fertility intention

Participants reported their number of living children, including with the partner with whom they were trying to conceive, as well as time frame of when they intended to conceive (within the next 0–6, 7–12,13–24 months). Respondents also indicated whether or not they had discussed their childbearing desires with their partner, and expressed a desire to talk with their HIV care providers. In addition, participants reported on their perception of their HIV provider’s willingness to discuss childbearing issues, and which provider would be suitable for the discussion. Whether the client or provider initiated the discussion content of discussion was reported.

#### Health management characteristics

Date of HIV diagnosis was self-reported, and CD4 count and ART status were abstracted from the participant’s clinic chart. Perceived quality of life was assessed by asking participants to respond to the question, “How has the quality of your life been during the past 30 days? That is, how have things been going for you?”, using a 1 ‘very good, could hardly be better’ to 5 ‘very bad, could hardly be worse’ response format; scores were reversed so that higher scores represent greater quality of life. To assess satisfaction with their HIV care, participants were asked to respond to the question “How satisfied are you with the HIV-related services provided at this clinic?” using a response format that ranged from 1 ‘very unsatisfied’ to 4 ‘very satisfied’.

#### Relationship and partner characteristics

These included marital status, whether respondent or partner had other spouses/partners (monogamous or polygamous relationship), HIV status of partner, and partner’s knowledge of respondent’s HIV status. Control of decision making in the relationship was measured with the 15-item relationship control subscale of the Sexual Relationship Power Scale [43]; respondents were asked to rate their level of agreement with statements from 1 ‘strongly agree’ to 4 ‘strongly disagree,’ a mean item score was calculated, and higher scores represent greater control in decision making within the relationship (Cronbach’s alpha = .82).

#### Psychosocial functioning

**Depression** was assessed using the 9-item Patient Health Questionnaire (PHQ-9) [44]; each item corresponds to the 9 symptoms assessed in the depression module of the Diagnostics Statistical Manual and is scored on a 0 ‘never’ to 3 ‘every day’ scale of symptom frequency over the past two weeks; the summary score is the sum of the item scores (Cronbach’s alpha = .82). Social support was measured with a single item from the ACTG assessment battery [45]; respondents rated their agreement with the statement, “I can count on my family and friends to give me the support I need” using a scale of 1 ‘strongly disagree’ to 5 ‘strongly agree’. Internalized HIV stigma was assessed with an 8-item scale developed by Kalichman et al. [46]; examples of items include “being HIV positive makes me feel damaged”, “I am ashamed that I am HIV positive”, “friends give me the support I need” and “I hide my HIV status from others”, with response options ranging from 1 ‘disagree strongly’ to 5 ‘agree strongly’ and a mean item score is calculated (Cronbach’s alpha = .75).

#### Stigma of childbearing among PLHIV

We developed a 2-item scale to measure the respondent’s internalized childbearing stigma: respondents were asked to indicate their level of agreement with the following statements, “I feel ashamed for wanting to have a child” and “I feel selfish for wanting to have a child”. Response options ranged from 1 ‘disagree strongly’ to 5 ‘agree strongly;’ mean item score was computed, and higher scores represent greater internalized childbearing stigma. We developed a single item to measure perceived provider childbearing stigma: respondents were asked to rate their level of agreement on a scale of 1 ‘disagree strongly’ to 5 ‘agree strongly’ with the statement, “Most HIV providers think that HIV-positive clients should not have children”.

### Data analysis

Descriptive statistics (frequencies, proportions, means, standard deviations, ranges) were used to describe demographic characteristics. Bivariate statistics [2-tailed, independent t-tests for continuous measures that were examined for normality (no transformations were required); Chi Square tests for binary or categorical measures] were used to examine cross-sectional correlates of client discussions with providers about childbearing intentions. Multivariate logistic regression analysis was used to further examine the correlates, with independent variables consisting of basic demographics (age, sex, any secondary education) and variables correlated with the dependent variable (communication with provider about childbearing intentions) in bivariate analysis at the p < .10 level of significance. Because of the exploratory nature of the analysis, we used P<0.10 instead of P<0.05.

## Results

### Sample characteristics

In total, 400 participants were enrolled (207 at Kampala, 193 at Jinja), the characteristics of whom are listed in Table 1. Three-quarters were female, and 61% were on ART. Less than half (44%) were married, but all others were in a committed relationship. Thirty per cent were in polygamous relationships, and all but one participant reported trying to conceive with just one partner. The majority (79%) reported that their partner was aware that the respondent was HIV-positive; however, nearly one-third (31%) of the respondents did not know their partner’s HIV status. Two-thirds (67%) reported that they planned to conceive within 6 months, 24% in 7–12 months from the time of the interview, and 9% within 13–24 months.

**Table 1:** Characteristics of people living with HIV desiring to have children.

Characteristics and other variables of the Sample (n = 400)	
Variable	Mean/Freq (SD or %)
***Demographics***	
Female	299 (74.8%)
Mean age (years)	33.8 (7.5)
Secondary education and above	179/379 (47.2%)
Operates a small business/sells things	194/399 (48.6%)
Salaried job	58/399 (14.5%)
Average monthly income $40-$220 USD	292/391 (74.7%)
***Health Characteristics***	
Mean years since HIV diagnosis	5.5 (4.7)
Mean CD4 count	435.4 (277.3)
On HIV antiretroviral therapy	242/399 (60.7%)
***Reproductive health history***	
Have had children	354 (88.5%)
Mean number of children (among parents)	3.2 (2.3)
Have had a child with current partner	195 (48.8%)
Had pregnancy since knowing HIV status	110/284 (38.7%)
Have had difficulty conceiving a child	135 (33.8%)
***Relationship/Partner Characteristics***	
Marital status:	
Married	175 (43.8%)
In committed relationship	225 (56.2%)
In a polygamous relationship	121 (30.3%)
HIV status of partner with whom trying to conceive	
HIV positive	156 (39%)
HIV negative	122 (30.5%)
Unknown HIV status	122 (30.5%)
Partner knows respondent’s HIV status	317 (79.3%)
***Childbearing desires*, *intentions and attitudes***	
Time frame for intending/planning to have a child	
(0–6 months)	267/399 (66.9%)
(7–12 months)	97/399 (24.3%)
(13–24 months)	35/399 (8.8%)
***Communication with providers about childbearing intentions***	
Have discussed their intentions with HIV providers	176 (44%)
Provider initiated the discussion on child bearing	50/176 (28.4%)
***Issues discussed with HIV providers***	
Discussed HIV transmission risk to partner	52/172 (30.2%)
Discussed HIV transmission risk to child	51/172 (29.7%)
Discussed information about PMTCT	46/172 (26.8%)
Discussed safer conception methods	14/172 (8.1%)
Discussion about treatment regimen	7/172 (4.1%)
HIV provider is willing to discuss childbearing	358/ 380 (94.2%)

### Communication with health providers about childbearing intentions

Nearly all (98%) respondents had both discussed having a child with their partner, and expressed a desire to discuss childbearing with their HIV provider, and 94% perceived that their HIV provider would be willing to discuss childbearing issues. Nonetheless, less than half (44%) had discussed their childbearing intentions with an HIV provider; women (46%) did not differ significantly from men (38%) in the discussion of childbearing intentions with a provider (Chi Square = 2.2, p = .135), nor did those who were married (41%) from those who were not (46%; Chi Square = 1.0, p = .310). When asked about the context in which they would like to discuss childbearing with providers, 95% preferred discussing childbearing with an HIV provider compared to a family planning provider (5%) or traditional healer (none); among providers at the HIV clinic, 60% preferred to discuss childbearing with a counselor, while 30% preferred to discuss childbearing with a doctor, and only 8% preferred a nurse.

Among those who had discussed childbearing, a minority (28%) reported that their provider had initiated the discussion. Among those who had discussed childbearing with their provider, the most common issues that were discussed included: HIV transmission risk to partner (30%), HIV transmission risk to child (30%), and information about prevention of mother-to-child transmission (PMTCT; 27%); only 8% discussed safer conception methods (Table 1).

### Correlates of communication with HIV providers about childbearing intentions

Table 2 lists the results of the bivariate correlates of communication with HIV providers about childbearing intentions. Greater time since HIV diagnosis (p <0.001), greater perceived quality of life (p <0.01), and having an HIV-positive partner (Chi square = 6.5, p<0.05) were significantly associated with having discussed childbearing intentions with a provider. Greater internalized HIV stigma (p< 0.001) and internalized childbearing stigma (p < .05) were both associated with not having discussed childbearing intentions with providers, and these two types of stigma were also correlated with each other (p < .001); perceived provider stigma of childbearing was unrelated to childbearing discussions with providers, but perceived provider stigma was positively correlated with internalized childbearing stigma (p < .001).

**Table 2:** Correlates of communication with HIV provider(s) about childbearing intentions.

Variable	Discussed with provider (n = 176)	Did not discuss with provider (n = 224)	Test Stat. (Chi-sqr. /*t* test)	*p* value
***Demographics***				
Age	33.7	33.8	0.193	0.847
Female Sex (%)	78.4	71.9	2.230	0.135
Has any secondary Education (%)	51.8	43.7	2.483	0.115
***Health Management***				
CD4 cell count	453	421	1.133	0.258
Current on ART (%)	63.1	58.7	0.771	0.380
Length of time since diagnosis (months)	78.4	56.3	3.936	<0.001
Satisfaction with HIV related services	3.80	3.80	1.930	0.587
Quality of life	3.8	3.6	3.174	0.002
***Relationship/Partner***				
Decision making power in relationship	2.61	2.59	0.316	0.753
Married (%)	40.9	46.0	1.031	0.310
In a polygamous relationship (%)	30.7	29.9	0.028	0.868
Has children (%)	86.4	90.2	1.409	0.235
Has children with conception partner (%)	47.2	50.0	0.318	0.573
Number of children	3.1	3.2	0.412	0.681
Partner is HIV positive (%)	46.0	33.5	6.515	0.011
Partner knows respondent’s HIV status (%)	83.5	75.9	3.489	0.062
***Psychosocial well-being***				
Depression	3.16	3.46	0.918	0.359
Social support	3.72	3.48	1.851	0.065
Internalized HIV stigma	2.13	2.40	3.378	0.001
***Childbearing Stigma***				
Internalized childbearing stigma	1.19	1.33	2.018	0.036
Perceived provider childbearing stigma	2.12	2.13	0.118	0.906

Table 3 shows the results of the multivariate logistic regression analysis of factors that influenced communication with HIV providers. Respondents with greater internalized childbearing stigma were less likely to discuss their childbearing intentions with providers [OR (95% CI) = 0.70 (0.49, .99)], while those who had communicated with providers about childbearing had been diagnosed with HIV for a longer period of time [OR (95% CI) = 1.09 (1.03, 1.15)]. The C statistic was equal to 0.662, suggesting that the model is a good fit to the data.

**Table 3:** Logistic regression of correlates of having discussed childbearing intentions with HIV provider(s).

*Variable*	OR (95% CI)	*p* value
***Background and Demographics***		
Age	0.99 (0.95, 1.03)	.544
Female Sex	1.46 (0.83, 2.13)	.193
Has any secondary education	1.38 (0.89, 2.17)	.152
Length of time since diagnosis (months)	1.09 (1.03, 1.15)	.002
***Relationship/Partner***		
Partner is HIV positive	1.32 (0.81, 2.13)	.261
Partner knows respondent’s HIV status	1.30 (0.70, 2.40)	.409
***Psychosocial well-being***		
Social support	1.14 (0.95, 1.36)	.153
Internalized HIV stigma	0.89 (0.65, 1.21)	.462
***Childbearing stigma***		
Internalized childbearing stigma	0.70 (0.49, .99)	.048

## Discussion

This study assessed client-provider communication about childbearing and its correlates among HIV infected individuals in care. While nearly all respondents expressed a desire to talk with their HIV provider about childbearing, less than half had done so. Our findings revealed that internalized stigma regarding both HIV and childbearing, but particularly regarding childbearing, were key barriers to communication between clients and providers about childbearing intentions. HIV clients who wanted to better understand how to prevent HIV transmission during attempts to conceive struggled to communicate with their health workers about childbearing intentions.

Although somewhat higher than the 20–40% reported in other studies [8, 27–29, 47, 48], our finding show that just under half of the sample had discussed their fertility intentions with providers, which reflects an unmet need for patient-provider communication about childbearing and HIV. Our data revealed that internalized stigma regarding childbearing may serve as a particularly key barrier to this communication. Internalized stigma regarding childbearing could be a result of experiences and perceptions of the judgmental attitudes of health workers, family and community who consider childbearing to be inappropriate for PLHIV [39, 40]. Similarly, provider stigma and judgmental attitudes amongst health workers has been shown to hinder provider discussion of safer conception methods with their clients [5, 23, 41, 44, 49, 50]. Studies have reported that childbearing stigma among both the providers and clients alike stifles both the content and depth of discussions about safer conception practices [27, 30–32]. Moreover, among respondents who discussed with providers, under one third talked about the risk of HIV transmission to partner, 30% discussed transmission to child and only 8% discussed safer conception methods. Conversely, other studies showed that low self-efficacy to provide SCC among HIV-providers could be a big contributor to the perceived stigma by clients [23, 24, 42]. It is therefore not surprising that among those who had discussed childbearing needs with providers, such communication was usually initiated by the client and not the provider, and these discussions rarely ever included instruction on how to use safer conception methods. However it is surprising that perceived provider stigma of childbearing was unrelated to client communication about childbearing needs with their provider, even though internalized childbearing stigma was positively correlated with perceived provider stigma.

Another form of internalized stigma, that being stigma associated with being HIV-infected, was also negatively correlated with having had discussed childbearing desires with providers in the bivariate analysis. Internalized HIV stigma has been identified as a key impediment to health seeking behaviors in other research [51, 52], so it is not surprising that stigma was a barrier to clients seeking childbearing support from providers, particularly if they perceive childbearing by PLHIV to be inappropriate or shameful. If a person feels shameful about their HIV status they may be more likely to believe that their desires for having a child are inappropriate because of their HIV status, as suggested by our data showing a correlation between HIV and childbearing internalized stigma, and thus less likely to discuss such desires with their providers.

Furthermore, our study shows that the clients who had known of their HIV status for a longer period of time were more likely to communicate with HIV providers about childbearing. Although not measured in this study, clients who had known their HIV diagnosis for a longer period of time may have also been in HIV care longer. This may imply that as the clients have more time to develop a rapport and to trust and become comfortable with their providers, they may be better able to overcome any perceived provider stigma and to communicate and articulate their childbearing desires. Similarly, for clients who have been in care longer, providers may interact with such clients in ways that reflect greater trust in and respect for client autonomy, which could lead to clients being comfortable to discuss fertility intentions [53].

Nearly all clients preferred discussing childbearing issues with HIV counselors, rather than family planning providers. In Uganda, this becomes challenging because HIV counselors are largely from social sciences background and have been trained to offer various kinds of HIV care but are not always grounded in other medical care including family planning. Furthermore, the current Ugandan family planning services for PLHIV have limited or no services for those clients who wish to conceive children.

Therefore, it becomes problematic when respondents prefer to discuss childbearing with counselors (rather than doctors or nurses) who typically are not medically trained and not conversant with fertility issues such as the timing of ovulation cycles. Counseling clients on childbearing and how to navigate components of family planning, including use of safer conception methods, warrants specialized training for health workers. However, this opportunity could be harnessed by training counselors to handle the initial childbearing discussions and sessions of navigating through risk reduction methods such as ART adherence, viral load suppression, STI screening and treatment which are imperative additions to other safer conception practices. Then, the counselors would refer the clients to doctors and nurses to handle the more medical methods of ovulation determination.

Clients rely on health providers for knowledge and guidance on how to conceive safely [3]. Moreover, nearly all participants in this study expressed a desire to discuss their childbearing needs with their providers, making it imperative that health workers become comfortable with and acquire the skills to counsel clients about safer conception and methods that can be used to promote safe childbearing. In our research with providers of HIV clients in Uganda, providers expressed a reluctance to discuss fertility desires with clients, despite a yearning to be able to provide safer conception counseling [23]. Provider reluctance to offer safer conception counseling was attributed to the absence of established policy guidelines, recommendations, training and counseling tools from the Ministry of Health for facilitating safer conception counseling.

### Limitations of the study

Although our analysis focused on communication between clients and providers, we relied solely on data from the client regarding whether such communication took place, as we had no data from the client’s provider regarding whether such communication took place. Furthermore, we relied on self-report, which is subject to recall and social desirability biases, rather than objective methods such as direct observation or audio-recordings. Other measurement limitations include our use of single item measures for constructs such as social support, provider stigma of childbearing, and satisfaction with care; use of more comprehensive, standardized measures would strengthen our ability to assess the relationships between these constructs and client communication of childbearing desires. In addition, our dataset did not have a variable to confirm clients’ own provider stigma as an influence to these discussions. However, the fact that clients responded with ‘the majority of HIV-care providers’ would suggest that their own HIV-care providers are alluded to. Lastly, communication is a behavior that may change over time. This paper reports baseline cross-sectional data, but when the study is completed we will be able to use the longitudinal data to assess whether communication between participants and their providers about childbearing intentions improved over time.

### Conclusions

In this sample of PLHIV with intentions to conceive, just less than half had discussed these intentions with their HIV providers. Most discussions about childbearing were initiated by clients, rather than their providers, and a minority included discussion of safer conception methods. Clients’ internalized childbearing stigma was a key barrier to their communicating with providers about childbearing intentions. Innovations to mitigate childbearing stigma among clients as well as training to improve health worker communication and family planning skills are critically needed. This would improve provider-client communication about family planning and especially safer conception among those that desire to conceive, and could help reduce transmission of HIV (in serodiscordant couples) or drug resistant viral strains (in concordant couples), as well as promote pregnancy and PMTCT (prevention of mother-to-child transmission) care management following conception.
